# Supplementary material for: Digestive Neurobiology in Autism: From Enteric and Central Nervous System Interactions to Shared Genetic Pathways
Source: Int J Mol Sci. 2025 Oct 1;26(19):9580. doi: 10.3390/ijms26199580 (PMC12525051; doi:10.3390/ijms26199580)
Supplement: Supplementary file 1 [file ijms-26-09580-s001.zip › Supplemental Figures (without tables).pdf]

- ## Supplemental Information

Supplemental Figure S1: Intersection between ASD-associated genes and genes expressed in the ENS. The list of 387 ASD-associated genes expressed *in the ENS.*

Number of common genes in ENS: 387

Common genes in ENS:

FBXL18, GRAMD1B, NRGN, FKBP9, MTHFD1L, KDM5D, NREP, SLC8A3, MAGI1, GPR26, PDE1C, PTPRN2, NPAS3, ANXA9, GALNT12, SEMA3E, KMT2D, MYO18A, KMT2A, ERLIN1, IPO9-AS1, MSL2, HLA-B, MSRA, DGKI, RPA3, AKAP6, HLA-DRB6, KMT2E, DPP6, ARHGAP15, GNAI1, ADAM22, MRPL33, RERE, CISD2, SYNGAP1, FOXP1, SEMA3A, RTN1, HLA-DMA, THSD4, ENOX1, GRIN2A, GIGYF2, FEZ1, CDHR4, PSMA5, VRK2, AGMO, PXDNL, RSRC1, RSU1, NCOA5, SPPL3, ESR2, MAPT, ESRRG, BANK1, WRN, UTY, TET2, GPR141, TCAIM, FADS2, TSNARE1, EFL1, KIZ, SSUH2, ZSCAN9, TMX2, DMXL2, PTCH1, PRKD1, PEF1, EXOC4, MDK, TYW1B, TMEM170B, PLCL1, DPP4, PKP4, SNX29, BBS9, MRM2, CYP2D7, SLC22A23, F2, LINC00461, PDE8B, SPAG16, CHRNA5, ZNF536, TAF1C, NPAS1, ANAPC4, TCF4, BEND4, EP300-AS1, CACNA2D2, DYNC1I1, ITIH3, CLSTN2, HFE, KCNG2, CTR9, USP4, MAST3, SBK1, KLC1, FHIT, SGF29, RBM6, NT5DC2, TNRC6A, MYO9B, CUL3, DOCK8, SLC6A9, CNTNAP2, PKD1L3, RPTOR, DPY19L1, PCLO, TRIM26, HDAC4, METTL13, PPM1E, L3MBTL4, SPNS1, TCF20, GABBR1, ZFPM2, BSN, MPHOSPH9, DYSF, CNTN4, C2-AS1, COL11A1, PFAS, OLFM4, THOC7, NEDD4L, CBLB, GPD2, SND1, SEMA6D, ZNF398, NR1D2, ANKS1B, FXR1, MMP16, ZNF823, EXT1, ITGA11, CALU, ATP2A2, ZMIZ1, DMTF1, ZNF804A, KIF5C, ARFGEF2, CITED1, DDX27, CKB, SFTA2, CHRNA3, CACNA1C, TTLL7, ZSCAN12, LDB1, GATB, ARID1B, EIF1AY, RAET1E, NT5C2, ZNF638, LINC01088, PRPF3, FGFR1, MSH5, HLA-DQA1, LRRC20, GLT8D1, CUEDC2, CNOT1, GUCY1A2, FTCDNL1, SLC13A1, PRKAG2, XRCC6, AUTS2, CASC15, AMIGO1, SLC30A9, SDK1, ETF1, CARMIL1, CREB5, ZSCAN31, MEF2C, ATP23, ZNF568, ZNF19, GMIP, GRIK1, DDX3Y, BTN2A1, MGAT5B, RHOJ, ZSWIM6, MAD1L1, DBN1, TFB1M, ZKSCAN3, MAGI2, CALD1, LINC01239, RASGRP4, CADM2, PALB2, CCT6P1, CTNND1, FSCN3, SNAP91, SOX5, CD55, IMPA2, CTNNB1, NEK4, ST3GAL3, NGEF, ADTRP, STAG1, NTM, SYT1, CACNB2, PCGF3, KCNB1, ANK3, PTPRF, ABCB9, FURIN, GUSBP2, MED27, DDN, PRKG1, CACNA1I, SRR, FBXL17, CTTNBP2, TWF2, CNNM2, LINC02057, PPP1R16B, LUZP2, ESRRB, MYBPHL, DHRS11, PLCL2, DPYD, CPEB1, TANK, IVD, CADPS, MICA, LRFN5, POM121C, MAML3, SLC35F2, BCL11A, ZCCHC7, ZSCAN16-AS1,

TSHZ3, NFIA-AS2, PSPH, ACTG1P17, FOXO3, RFT1, LCORL, PTPRO, CLEC17A, KDM4A, JMJD1C, TMEM219, RFC2, ZNF600, GBF1, MEF2C-AS1, PIP5K1B, HLA-DRB5, KEL, PDE4B, NKIRAS1, SHISA9, SEMA3F, RNF111, MIRLET7BHG, KCNN2, RBL2, PMFBP1, LONRF2, GID4, POLR2E, ZFYVE1, GLCCI1, SORCS3, LINC01876, BTN3A2, ZMIZ2, PARD3B, PPP2R2B, HLA-DRB1, SEMA3F-AS1, RGS6, PPP1R13B, TENM2, CCDC88C, MANBA, SGSM2, STARD4-AS1, NFIX, SRPK2, NTRK3, GALNT1, TNXB, NEGR1, SYNE1, GALNT10, GATAD2B, HLA-DQB1, PPP1R16A, ZNF615, ELMO1, DPP8, NOVA1, IMMP2L, WNT3, PCDH9, THRB, MACROD2, CLCN3, SLC39A8, PPA2, PREX1, ATP2B2, LINC01572, RPS6KL1, BTN2A2, PPP2R5C, KIF21B, BRWD1, TENM4, DOCK4, FAM193A, TRAIP, IGSF9B, KDM3B, TRIM33, SORBS1, LINC00240, BCL11B, FOXO6, AMBRA1, CSRNP3, NDUFA2, MED8, GLI3, ZKSCAN8, NEURL1, RHOA, PIK3R2, DNAH11, TMOD3, BTN3A1, AFF3, PUS7, TRIOBP, NAALADL2, HDAC9, MAIP1, RNF144A, CAMKV, ZNF800, CDKAL1, TLK1, SREK1IP1

Supplemental Figure S2: Molecular function, cellular component, and biological pathway analysis for ASD-associated genes expressed in the ENS, without pathways with FDR<0.05. a. Molecular function analysis with the top-enriched GO functions. **b.** Molecular function analysis with the top-enriched GO functions. **c.** Biological pathway analysis with the top-enriched Reactome pathways. **d.** Biological pathway analysis with the top-enriched KEGG pathways.

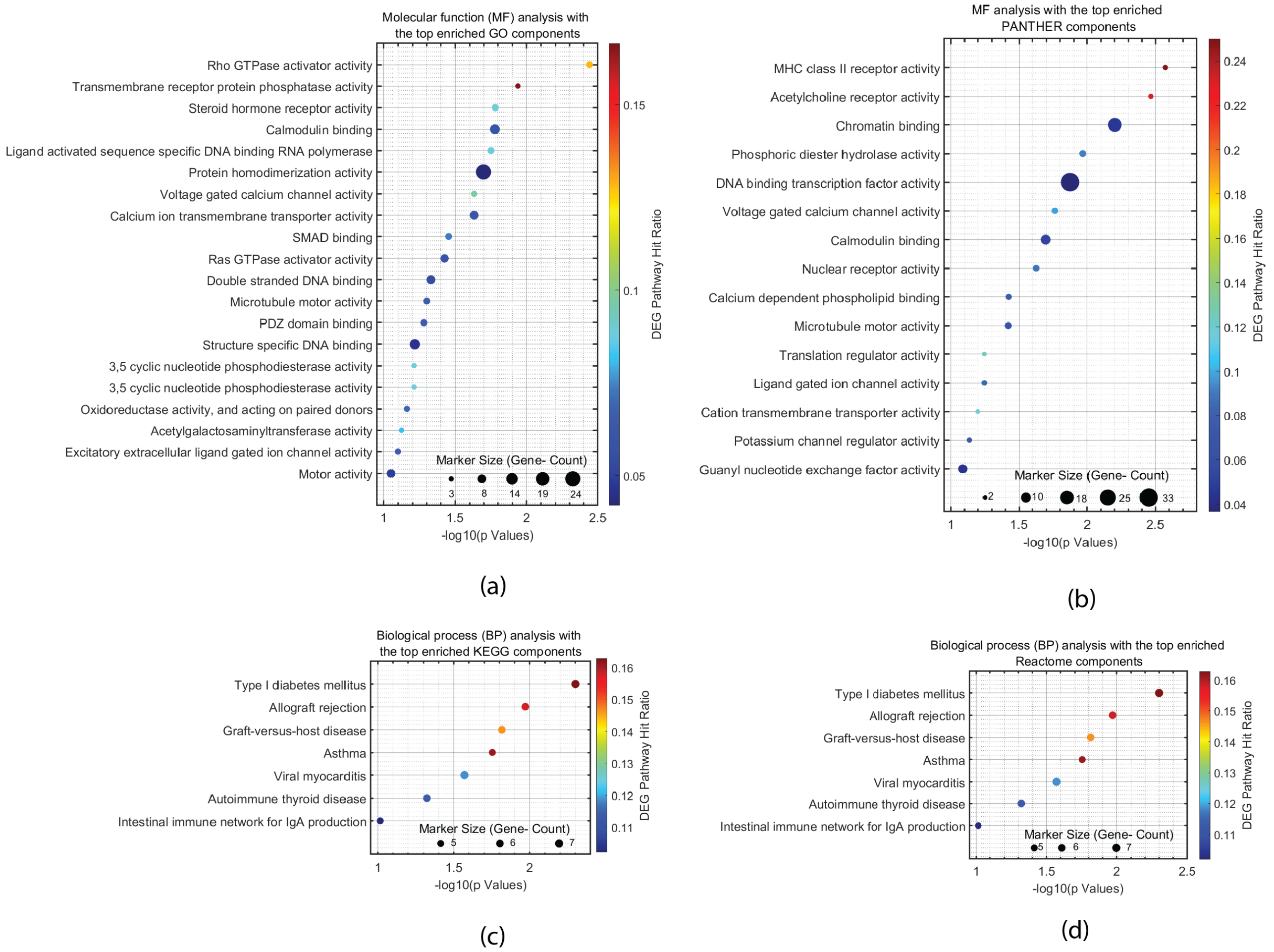

Supplemental Table S1: GO-Enriched Biological Pathway

Supplemental Table S2: GO-Enriched Cell Components

Supplemental Table S3: GO-Enriched Molecular Functions

Supplemental Table S4: PANTHER-Enriched Biological Pathways

Supplemental Table S5: PANTHER-Enriched Cell Components

Supplemental Table S6: PANTHER-Enriched Molecular Functions

Supplemental Table S7: KEGG-Enriched Biological Pathways

Supplemental Table S8: Reactome-Enriched Biological Pathways

Supplemental Table S9: Motif-Enriched
